# Supplementary material for: Direct correction of haemoglobin E β-thalassaemia using base editors
Source: Nat Commun. 2023 Apr 19;14:2238. doi: 10.1038/s41467-023-37604-8 (PMC10115876; doi:10.1038/s41467-023-37604-8)
Supplement: Supplementary file 1 — Supplementary Information [file 41467_2023_37604_MOESM1_ESM.docx]

**Supplementary Information**

**Index of Supplementary Information**

|  | Page |
| --- | --- |
| Index | 1 |
| Supplementary Fig. 1 | 2 |
| Supplementary Fig. 2 | 3 |
| Supplementary Fig. 3 | 4 |
| Supplementary Fig. 4 | 5 |
| Primers and gRNA sequences | 6 |
| Flow cytometry gating strategy | 7 |
| Supplementary Data Table Legends | 8 |


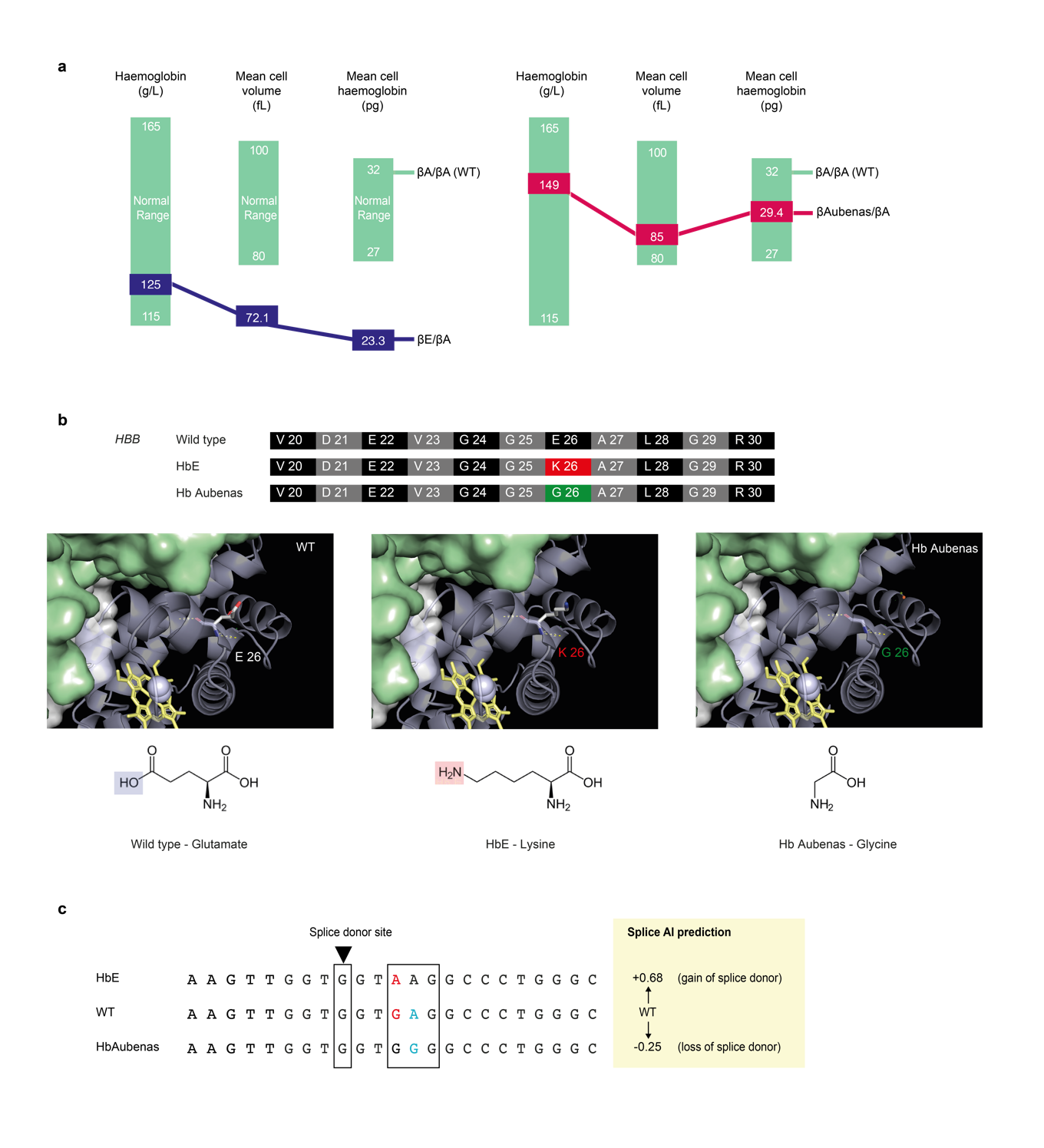


**Supplementary Fig. 1** **a,** Comparison of blood count data from a patient with HbE trait^36^ compared to Hb Aubenas, showing that Hb Aubenas confers normal blood count indices. Two other family members also had asymptomatic carriage^18^. **b,** Structure of the WT, HbE and Hb Aubenas variants. HbE results from a substitution of glutamate, a negatively charged side chain, to lysine, which is positively charged. By contrast the glutamate to glycine substitution in Hb Aubenas is unlikely to be pathogenic as it creates a third consecutive glycine an alpha helical domain. **c,** Prediction of splicing effects using Splice AI showing that HbE is predicted to create a cryptic splice site whereas HbAubenas has lower aberrant splicing potential compared to the canonical sequence^15^.


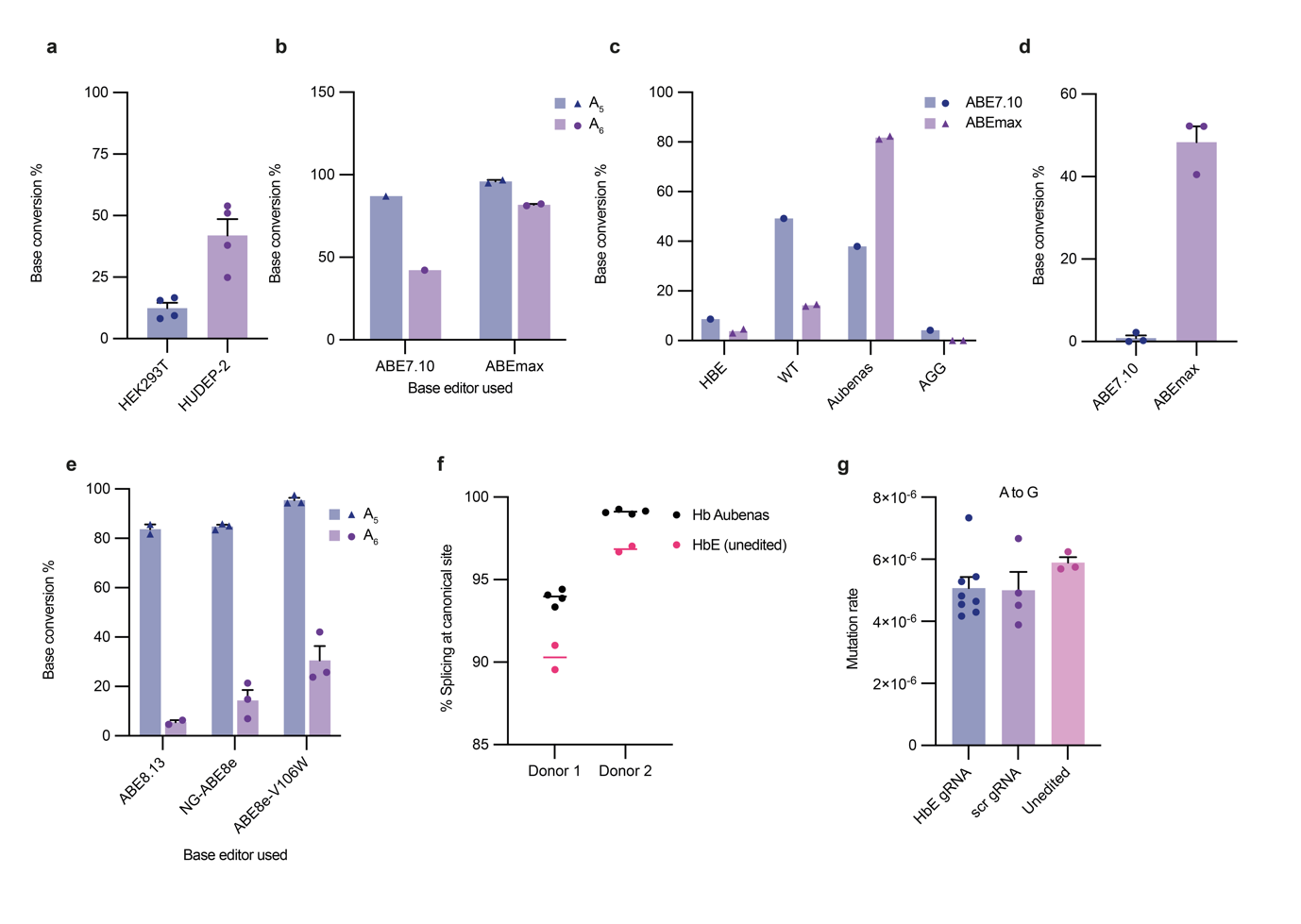


**Supplementary Fig. 2 Optimisation of base editing at HbE a,** Initial optimisation was carried out using plasmid transfection in WT HEK293T and HUDEP-2 cells. Editing at A_6_ was more efficient in HUDEP-2 cells **b,** editing with the constructs ABE7.10 and ABEmax were tested in a HUDEP-2 line engineered with the HbE mutation in homozygosity. Both have high on-target A_5_ editing efficiency with ABEmax having increased editing efficiency at the bystander base A_6_. **c,** codon outcomes as identified by NGS when editing the HbE line with ABE7.10 and ABEmax plasmid. ABEmax has higher on-target and bystander editing, resulting in a higher number of reads with the Aubenas codon. **d,** editing in WT human primary CD34+ cells using ABE7.10 and ABEmax plasmid. Editing at A6 was poor with ABE7.10 but much more efficient using ABEmax, likely due to the latter’s improved codon usage and nuclear localisation signals. **e,** editing in HbE/β-thalassaemia patient cells with mRNA transcripts for ABE8.13, ABE8e-V106W and NG-ABE8e. The most efficient editor was consistently ABE8e-V106W. **f,** Splicing assessed by RNA-seq at the canonical splice site is increased following editing to HbAubenas compared to unedited HbE. (P < 0.0001 Two-Way ANOVA). g, Comparison of frequency of A to G transitions in RNA-seq data from day 10 erythroid cells following base editing with ABE8e-V106W. No significant persistent editing was seen (P – 0.43 One-way ANOVA). All error bars represent the standard error of the mean.


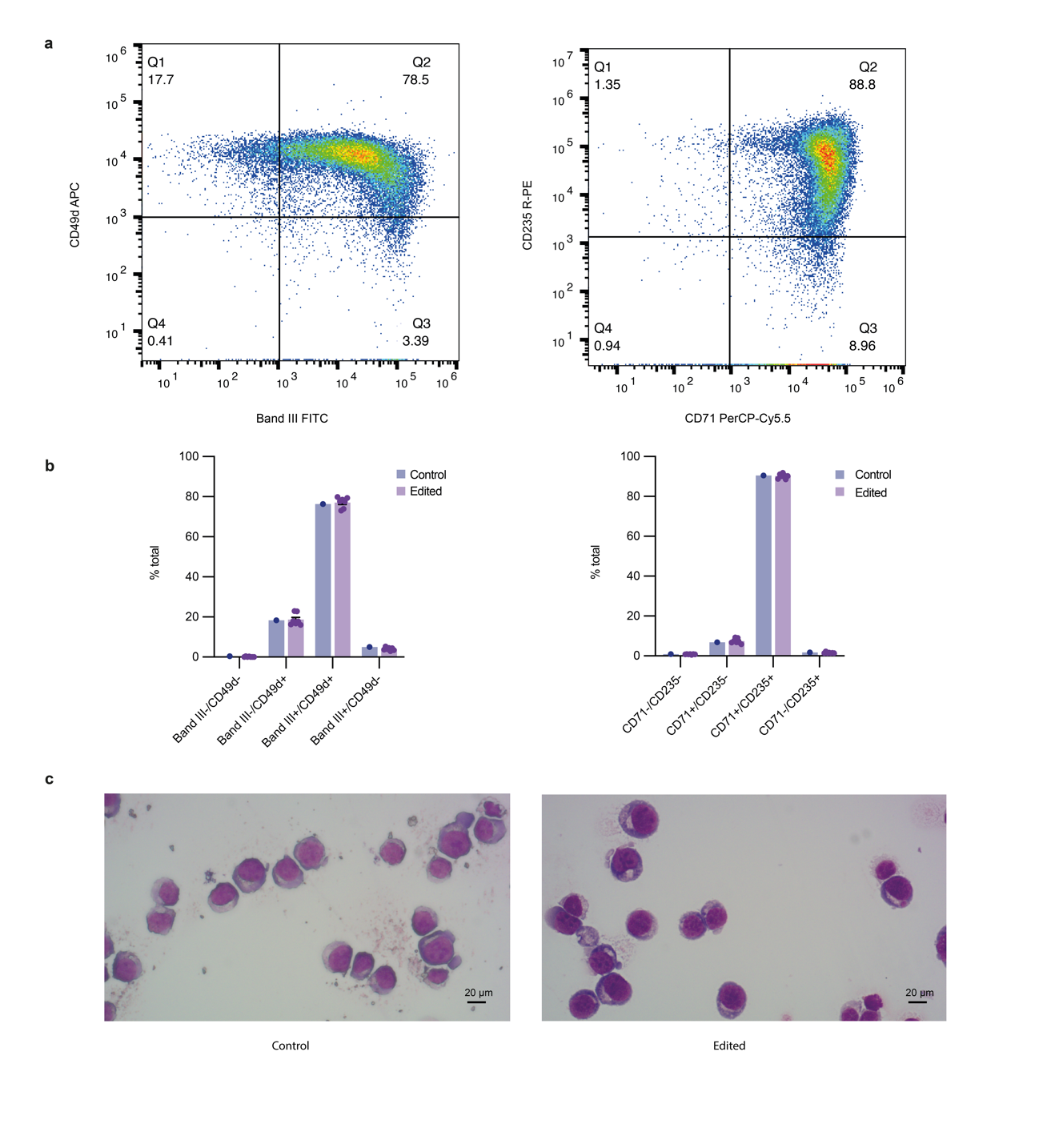


**Supplementary Fig. 3 a&b,** Representative flow cytometry data showing erythroid maturation at day 10 of differentiation in edited cells from a single donor (see Supplementary material for gating strategy). Scrambled sgRNA control transfected cells (n=1) were at the same stage of differentiation as edited cells (n=7). **c,** Representative cytospins at day 10 of differentiation. Cytospins were made for the control and each of the edited samples shown in Supplementary Fig. 3 a&b with similar results (n=8). Left panel Scr control transfected sample, right panel edited sample. Stained with modified Wright’s stain, x40 magnification NTC, non-transfection control; scr, scrambled sgRNA. All error bars represent the SEM.


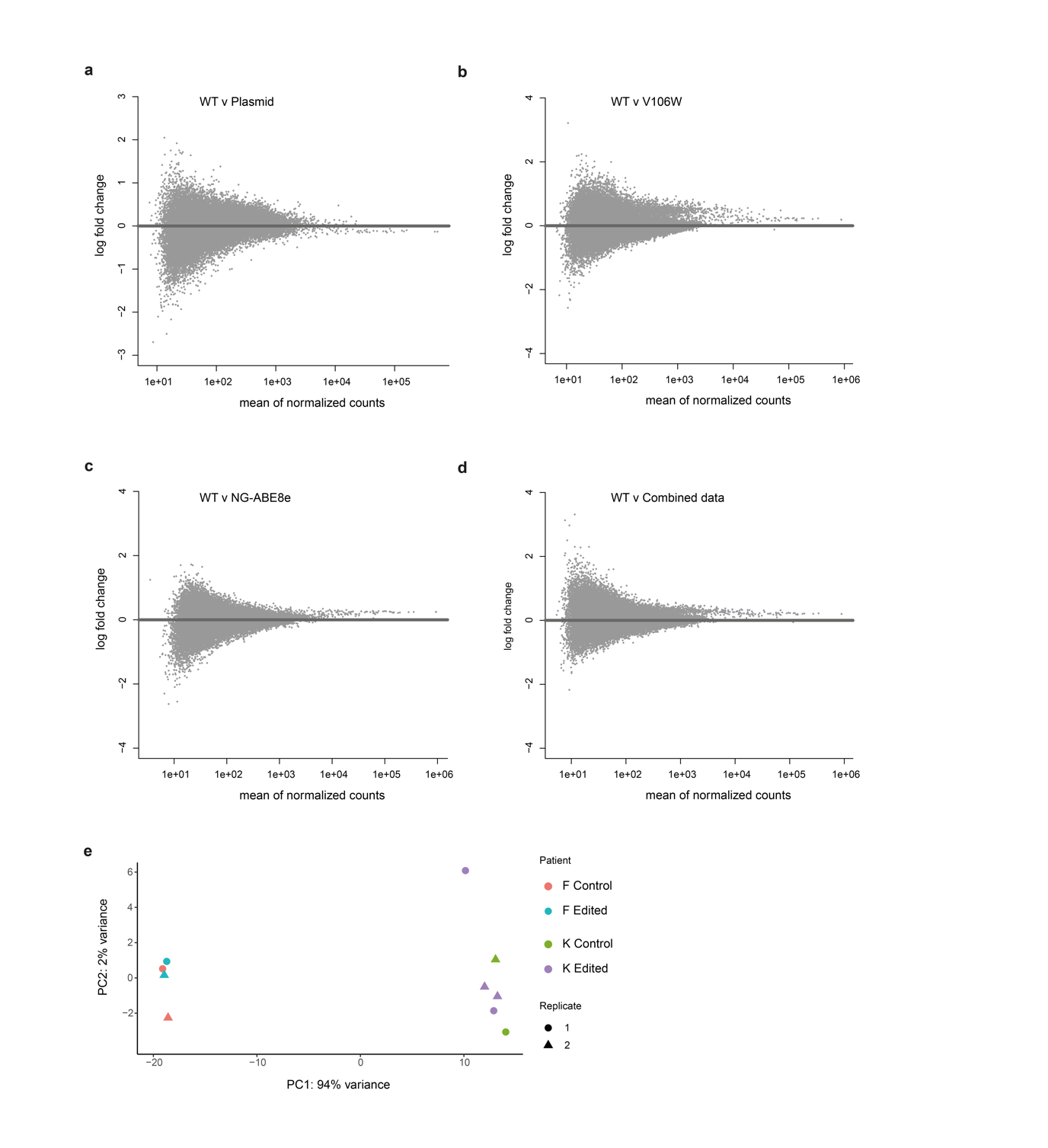


**Supplementary Fig. 4 Global chromatin accessibility profiles MA plots showing a lack of differentially accessible peaks between edited and non-edited patient samples a,** plasmid-based editing **b,** mRNA based editing of cells using V106W and **c,** NG-ABE8e. **d,** comparison combining all data from **b&c. e,** PCA of dataset combining inputs from two patients, showing the majority of variation derives between biological replicates rather than between control and edited samples. (DESeq2, alpha 0.05).

**Supplementary Methods**

**Primers and gRNA sequences**

Primers for amplification of HBB gene

Forward primer:

5’ GTAAACGACGGCCAGTTTAGACCTCACCCTGTGGAGC 3’

Reverse primer:

5’ GACTGGAGTTCAGACGTGTGCTCTTCCGATCTCAAAGGACTCAAAGAACCTC 3’

gRNA sequences

HbE gRNA

5’ UGGUAAGGCCCUGGGCAGGU 3’

Wild type gRNA

5’ UGGUGAGGCCCUGGGCAGGU 3’

**Flow cytometry gating strategy**


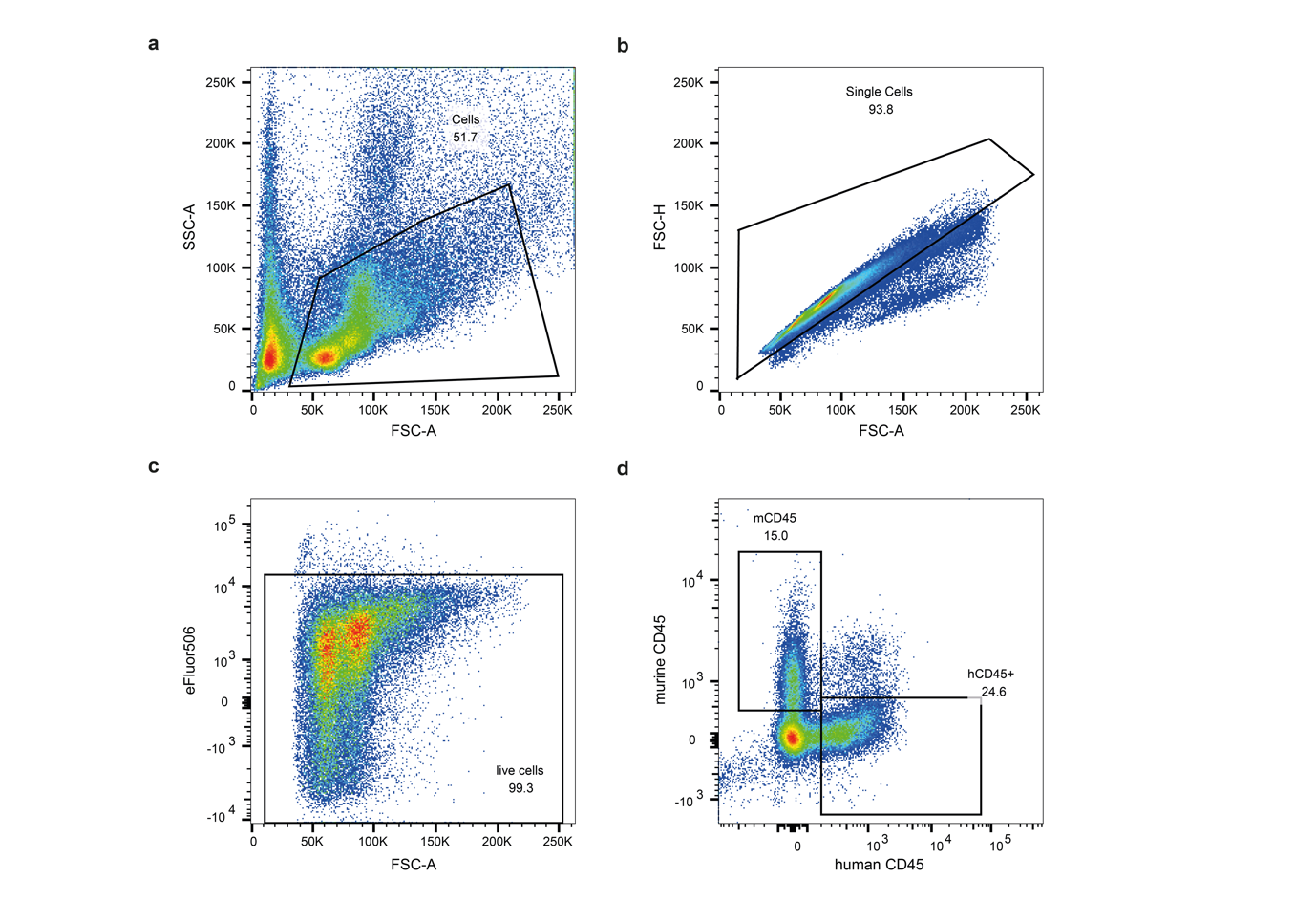


Gating strategy for analysis of primary and secondary xenotransplants

**Antibodies**

CD235a PE - BD Bioscience 555570

CD71 PerCP Cy5.5 Biolegend 334114

CD49D APC BD Bioscience 861392

CD34 PE/Cy Biolegend 343616

CD233 FITC IBGRL 9439

CD36 APC/Cy7 Biolegend 336213

Hoechst 33258 Invitrogen H3569

Fixable Viability Dye eFluor 506 eBioscience 65-0866-14

mCD45 eBioscience 48-0451-82

hCD45 Invitrogen MHCD4505

CD3 eBioscience 47-0038-42

CD235a eBioscience 17-9987-42

CD34 eBioscience 12-0349-42

CD19 Biolegend 302208

CD33 eBioscience 25-0338-42
