# Supplementary material for: Direct correction of haemoglobin E β-thalassaemia using base editors
Source: Nat Commun. 2023 Apr 19;14:2238. doi: 10.1038/s41467-023-37604-8 (PMC10115876; doi:10.1038/s41467-023-37604-8)
Supplement: Supplementary file 3 — Description of Additional Supplementary Files [file 41467_2023_37604_MOESM3_ESM.docx]

**Description of Additional Supplementary Files**

**File Name: Supplementary Data 1.**

**Description: Potential off-target sites**

Potential off-target sites were initially identified using a combination of CIRCLE-seq, which profiles the *in vitro* activity of gRNAs and *in silico* prediction tools, which included CRISPOR and Cas-OFFinder. Potential off-target sites were annotated using HOMER (v4.7)

**File Name: Supplementary Data 2.**

**Description: Capture oligo design**

Oligonucleotides were designed to capture the top 250 sites identified by the combination of CIRCLE-seq and in silico methods. These 120mer sequences were designed using CapSequm.

**File Name: Supplementary Data 3.**

**Description: Results of targeted oligonucleotide capture**

Analysis of targeted capture data showing frequency of variants within the predicted base editing window at the sites identified in Supplementary Data Table 1. The columns include the reference allele, alternate allele, depth of sequencing and number of sequenced mutations across all samples (n=7 for edited samples n=1 for controls).

**File Name: Supplementary Data 4.**

**Description: Data sets used for machine learning predictions of functional off-target effects**

38 different classifiers from the deep neural network were used for prediction of functional off-target effects.

**File Name: Supplementary Data 5.**

**Description: Results of machine learning analysis of functional off-target sites.**

All potential off-target sites were analysed and ordered based on likelihood of damaging or creating a new regulatory sequence in the non-coding genome. A positive damage score indicates destruction of a regulatory element whereas a negative score indicates creation of a new element.

**File Name: Supplementary Data 6.**

**Description: Genotypes of the patients included in the study**
