# Supplementary material for: Direct correction of haemoglobin E β-thalassaemia using base editors
Source: Nat Commun. 2023 Apr 19;14:2238. doi: 10.1038/s41467-023-37604-8 (PMC10115876; doi:10.1038/s41467-023-37604-8)
Supplement: Supplementary file 10 — Reporting Summary [file 41467_2023_37604_MOESM10_ESM.pdf]

nature portfolio

Corresponding author(s): James Davies

Last updated by author(s): 23/1/23

Reporting Summary

Nature Portfolio wishes to improve the reproducibility of the work that we publish. This form provides structure for consistency and transparency in reporting. For further information on Nature Portfolio policies, see our Editorial Policies and the Editorial Policy Checklist.

Statistics

For all statistical analyses, confirm that the following items are present in the figure legend, table legend, main text, or Methods section.

n/a	Confirmed	
☐	☒	The exact sample size (n) for each experimental group/condition, given as a discrete number and unit of measurement
☒	☐	A statement on whether measurements were taken from distinct samples or whether the same sample was measured repeatedly
☐	☒	The statistical test(s) used AND whether they are one- or two-sided<br>*Only common tests should be described solely by name; describe more complex techniques in the Methods section.*
☒	☐	A description of all covariates tested
☒	☐	A description of any assumptions or corrections, such as tests of normality and adjustment for multiple comparisons
☐	☒	A full description of the statistical parameters including central tendency (e.g. means) or other basic estimates (e.g. regression coefficient) AND variation (e.g. standard deviation) or associated estimates of uncertainty (e.g. confidence intervals)
☐	☒	For null hypothesis testing, the test statistic (e.g. F, t, r) with confidence intervals, effect sizes, degrees of freedom and P value noted<br>*Give P values as exact values whenever suitable.*
☒	☐	For Bayesian analysis, information on the choice of priors and Markov chain Monte Carlo settings
☒	☐	For hierarchical and complex designs, identification of the appropriate level for tests and full reporting of outcomes
☒	☐	Estimates of effect sizes (e.g. Cohen's d, Pearson's r), indicating how they were calculated

Our web collection on statistics for biologists contains articles on many of the points above.

Software and code

Policy information about availability of computer code

Data collection	Illumina Miseq control software v 4.0 was used on the Illumina Miseq to collect the NGS sequencing data for editing outcomes.

Data analysis	All custom scripts are available on GitHub (https://github.com/jojdavies/Base_editing_off-targets)<br>Bowtie 2 (v2.3.5)<br>Base editing data was analysed using CRISPResso (V2 https://github.com/pinellolab/CRISPResso2)<br>CasOFFfinder (2.4 http://www.rgenome.net/cas-offinder/)<br>CRISPOR (5.01)<br>circleseq Python package (1.1 https://github.com/tsailabSJ/circleseq)<br>FLASH (v1.2.11)<br>FlowJo (10.8.1)<br>GATK (4.0.11.0)<br>MACS2 (v2.0.10)<br>DESeq2 (v3.12)<br>Prism (9.5.0)<br>PySam (0.20.0) (https://github.com/pysam-developers/pysam)<br>RStudio (1.2.5033)<br>Samtools (1.10)<br>STAR (2.7.3a)<br>Trim Galore (Babraham Instiitute, v0.3.1)

For manuscripts utilizing custom algorithms or software that are central to the research but not yet described in published literature, software must be made available to editors and reviewers. We strongly encourage code deposition in a community repository (e.g. GitHub). See the Nature Portfolio guidelines for submitting code & software for further information.

Data

Policy information about availability of data

All manuscripts must include a data availability statement. This statement should provide the following information, where applicable:

- Accession codes, unique identifiers, or web links for publicly available datasets
- A description of any restrictions on data availability
- For clinical datasets or third party data, please ensure that the statement adheres to our policy

Sequencing data has been submitted to the NCBI Gene Expression Omnibus (GSE206098)

Human research participants

Policy information about studies involving human research participants and Sex and Gender in Research.

Reporting on sex and gender	Findings are expected to apply equally to males and females
Population characteristics	All patients had HbE/beta-thalassaemia, with five of six being transfusion dependent.
Recruitment	Patients were recruited from the clinics of the Oxford University Hospitals Trust, Barts Health NHS trust and University of Kelaniya & Colombo North Teaching Hospital. The patients were solely recruited by the attending physicians and they were felt to be reflective of the general patient cohort by the attending physician. Patients received no immediate benefit from partaking in the study, however, self-selection bias was not expected to impact the results of the study as cells were removed from patients with the same genotype and edited at a distant site.
Ethics oversight	The study has approval from the Oxford South Central C Research Ethics Board; WIMM R&D committee (ref. 17/SC/0111) and the Sri Lanka College of Paediatricians. Human umbilical cord blood (UCB) was collected from the John Radcliffe Hospital, Oxford, UK or provided via the NHS Cord Blood Bank, London, and used with informed, written pre-consent and ethical approval (REC Ref. no. 15/SC/0027) from the South Central Oxford and Berkshire Ethical Committees and approval of the NHSBT R&D committee.

Note that full information on the approval of the study protocol must also be provided in the manuscript.

Field-specific reporting

Please select the one below that is the best fit for your research. If you are not sure, read the appropriate sections before making your selection.

[x] Life sciences [ ] Behavioural & social sciences [ ] Ecological, evolutionary & environmental sciences

For a reference copy of the document with all sections, see nature.com/documents/nr-reporting-summary-flat.pdf

Life sciences study design

All studies must disclose on these points even when the disclosure is negative.

Sample size	Sample sizes were not determined prior to the experiment, but were based on the availability of patient-cells.
Data exclusions	Data were not excluded
Replication	All experiments were undertaken in at least biological triplicates from different donors. The number of samples analysed differed slightly between experiments and full details are included in the manuscript. For the key experiments delineating editing efficiencies 6 biological replicates were included.
Randomization	Randomization was not required for the editing experiments because it was possible to split the cells from each donor were so that we had an internal control for each sample.
Blinding	Mouse experiments were blinded. Mice were kept by staff at the animal facility without knowledge of which samples had been injected into which mice, whether primary or secondary xenotransplant.

Reporting for specific materials, systems and methods

We require information from authors about some types of materials, experimental systems and methods used in many studies. Here, indicate whether each material, system or method listed is relevant to your study. If you are not sure if a list item applies to your research, read the appropriate section before selecting a response.

Materials & experimental systems

n/a	Involved in the study
☐	☒ Antibodies
☐	☒ Eukaryotic cell lines
☒	☐ Palaeontology and archaeology
☐	☒ Animals and other organisms
☒	☐ Clinical data
☒	☐ Dual use research of concern

Methods

n/a	Involved in the study
☒	☐ ChIP-seq
☐	☒ Flow cytometry
☒	☐ MRI-based neuroimaging

Antibodies

Antibodies used	CD235a PE - BD Bioscience (555570 GA-R2 (HIR2))<br>CD71 PerCP Cy5.5 Biolegend (334114 CY1G4)<br>CD49D APC BD Bioscience (861392 9F10)<br>CD34 PE/Cy Biolegend (343616 561)<br>CD233 FITC IBGRL (9439 BRIC 6)<br>CD36 APC/Cy7 Biolegend (336213 5-271)<br>Hoechst 33258 Invitrogen (H3569)<br>Fixable Viability Dye eFluor 506 eBioscience (65-0866-14)<br>mCD45 eBioscience (48-0451-82 30-F11)<br>hCD45 Invitrogen (MHCD4505 HI30)<br>CD3 eBioscience (47-0038-42 UCHT1)<br>CD235a eBioscience (17-9987-42 GA-R2 (HIR2))<br>CD34 eBioscience (12-0349-42 4H11)<br>CD19 Biolegend (302208 HIB19)<br>CD33 eBioscience (25-0338-42 WM-53)
Validation	All antibodies have been validated by the manufacturers and were titrated in house on primary human cells.

Eukaryotic cell lines

Policy information about cell lines and Sex and Gender in Research

Cell line source(s)	HUDEP-2 cells were kindly provided by Dr Kurita and Dr Nakamura from the RIKEN Tsukuba Branch.
Authentication	Cells karyotyped by Infinium Omni5-4 v1.2 CGH array (Moir-Meyer et al, Methods Protoc. 2018)
Mycoplasma contamination	These cells are regularly tested for mycoplasma contamination (every 1-2 months). No contamination was detected in any of the cells used in these experiments.

Commonly misidentified lines (See ICLAC register)	None used

Animals and other research organisms

Policy information about studies involving animals; ARRIVE guidelines recommended for reporting animal research, and Sex and Gender in Research

Laboratory animals	Mouse, NOD.Cg-PrkdcscidIl2rgtmlWjl/SzJ (Jackson laboratory). All experimental animals were 8–12-week-old female mice. They were housed in individually ventilated cages, which were enriched with red tunnels or houses and balconies. They were kept with 12-h light/dark cycle at 21–22 °C and 45–65% relative humidity.
Wild animals	No wild animals were included in the study
Reporting on sex	Female mice were utilised for xenotransplants due to superior engraftment of primary human cells.
Field-collected samples	No field collected samples were included in the study
Ethics oversight	Experiments were performed under the project license P8869535A approved by the Oxford University Clinical Medicine Ethical Review committee and the UK Home Office under the Animal (Scientific Procedures) Act 1986.

Note that full information on the approval of the study protocol must also be provided in the manuscript.

Flow Cytometry

Plots

Confirm that:

- [x] The axis labels state the marker and fluorochrome used (e.g. CD4-FITC).
- [x] The axis scales are clearly visible. Include numbers along axes only for bottom left plot of group (a 'group' is an analysis of identical markers).
- [x] All plots are contour plots with outliers or pseudocolor plots.
- [x] A numerical value for number of cells or percentage (with statistics) is provided.

Methodology

Sample preparation	CD34+ cells were resuspended in Phase I medium for a three-phase differentiation adapted from that used for the BEL-A cell line (Trakarnsanga, Nat Comms 2017). Briefly, for differentiation 3x10^5 cells were resuspended on day 0 in Phase I media at 2x105 cells ml-1. Cell counts were performed on days 3 and 5 with additional Phase I media added to return the concentration to 105 cells ml-1. On day 7, cells were counted and pelleted (300 rcf, 5 min, RT) and resuspended in Phase II media at 2x105 cells ml-1. Cells were counted on day 9 and diluted to 2x105 cells ml-1 Phase II media. HUDEP-2 cells were cultured in expansion phase as previously described Canver, M. C. et al.,2015.
Instrument	Flow cytometry was performed on the Attune NxT Flow cytometer (Invitrogen). 10.7.1).
Software	Data was analysed on FlowJo software (V. 10.7.1)
Cell population abundance	Cell abundance was determined by flow cytometric analysis of cells. No sorting was conducted.
Gating strategy	FSC-A/SSC-A for mononuclear cells.<br>SSC-A/SSC-W for doublets<br>Hoechst 33258 for viability<br>Anti-human CD235 PE<br>Anti-human CD71 PerCP Cy5.5<br>Of the CD235+ cells:<br>Anti-human CD49d APC<br>Anti-human Band III (CD233) FITC

- [x] Tick this box to confirm that a figure exemplifying the gating strategy is provided in the Supplementary Information.
